# VarioML framework for comprehensive variation data representation and exchange

**DOI:** 10.1186/1471-2105-13-254

**Published:** 2012-10-03

**Authors:** Myles Byrne, Ivo FAC Fokkema, Owen Lancaster, Tomasz Adamusiak, Anni Ahonen-Bishopp, David Atlan, Christophe Béroud, Michael Cornell, Raymond Dalgleish, Andrew Devereau, George P Patrinos, Morris A Swertz, Peter EM Taschner, Gudmundur A Thorisson, Mauno Vihinen, Anthony J Brookes, Juha Muilu

**Affiliations:** 1 Institute for Molecular Medicine Finland (FIMM), University of Helsinki, Helsinki, Finland; 2 Department of Human Genetics, Leiden University Medical Center, Leiden, Netherlands; 3 Department of Genetics, University of Leicester, Leicester, UK; 4 Medical College of Wisconsin, Milwaukee, WI, USA; 5 Biocomputing Platforms, Ltd, Espoo, Finland; 6 Phenosystems Inc, Brussels, Belgium; 7 INSERM UMR_S910, Faculté de Médecine La Timone, Marseille, France; 8 National Genetics Reference Laboratory, Manchester, UK; 9 Department of Pharmacy, School of Health Sciences, University of Patras, Patras, Greece; 10 Department of Genetics, Genomics Coordination Center University Medical Center Groningen and Groningen Bioinformatics Center, University of Groningen, Groningen, Netherlands; 11 Department of Experimental Medical Science, Lund University, Lund, Sweden; 12 Institute of Biomedical Technology, University of Tampere, Tampere, Finland; 13 Tampere University Hospital, Tampere, Finland

**Keywords:** LSDB, Variation database curation, Data collection, Distribution

## Abstract

**Background:**

Sharing of data about variation and the associated phenotypes is a critical need, yet variant information can be arbitrarily complex, making a single standard vocabulary elusive and re-formatting difficult. Complex standards have proven too time-consuming to implement.

**Results:**

The GEN2PHEN project addressed these difficulties by developing a comprehensive data model for capturing biomedical observations, Observ-OM, and building the VarioML format around it. VarioML pairs a simplified open specification for describing variants, with a toolkit for adapting the specification into one's own research workflow. Straightforward variant data can be captured, federated, and exchanged with no overhead; more complex data can be described, without loss of compatibility. The open specification enables push-button submission to gene variant databases (LSDBs) e.g., the Leiden Open Variation Database, using the Cafe Variome data publishing service, while VarioML bidirectionally transforms data between XML and web-application code formats, opening up new possibilities for open source web applications building on shared data. A Java implementation toolkit makes VarioML easily integrated into biomedical applications. VarioML is designed primarily for LSDB data submission and transfer scenarios, but can also be used as a standard variation data format for JSON and XML document databases and user interface components.

**Conclusions:**

VarioML is a set of tools and practices improving the availability, quality, and comprehensibility of human variation information. It enables researchers, diagnostic laboratories, and clinics to share that information with ease, clarity, and without ambiguity.

## Background

The study of disease-causing and benign variations in the human genome is progressing rapidly. Whole genome and exome sequencing continues to expand, and improved tools for variant calling are becoming available [1-3]. Cost-effective sequencing, paired with variant discovery, promises to make early detection and intervention accessible for the millions of individuals with genetic diseases.

However, realizing this potential is blocked by the problem of integrating and coordinating the steps towards “a pipeline leading from discovery to delivery” [4]. The GEN2PHEN project was initiated in 2008 to unify human and model organism genetic variation databases, and remove the obstacles to translation of variant data from laboratory to clinic to public [5]. This has involved attempting to unify the divergent data representations of various database communities.

The focus of this effort is the locus-specific database (LSDB) [6]. LSDBs describe the variants discovered on a single gene, a gene family or a group of genes involved in the similar diseases or traits. As of this writing, 4,111 LSDBs can be easily searched online [7]. LSDBs are curated by experts on their respective loci, and as such are typically the best resources of gene variant information available [8]. A comprehensive 2010 analysis of 1,188 LSDBs provides a useful overview of the domain, providing encouraging results, such as finding only 5.4% to be outdated [9]. However, the study also found that only 8% provided detailed disease and phenotypic descriptions. LSDBs also vary widely in format, diverging to satisfy the immediate requirements of numerous use cases, making comprehensive, global analysis of data concerning a given variant difficult, if not impossible [10]. LSDBs are also typically incomplete, either from a lack of capacity on the part of the data submitters or curators to include all pertinent data, or from the original data lacking key elements altogether [9,11]. It is well recognised that the data will often be incomplete if you ask too much of the submitters [12]. Into this situation, next-generation sequencing pipelines are rapidly increasing the scale and complexity of data to be managed [13].

## Methods

### Undesigning a standard

All terms and abbreviations used are explained in the glossary (Table 1).

**Table 1:** Glossary

**Name**	**Definition**	**URL**
API	Application programming interface	*-*
BSVM	Pioneering early LSDB integration standard.	See *Tyrelle G, King GC, 2003*[15]
Café Variome	Variation data publishing service	*http://cafevariome.org/*
Extended Backus-Naur Form	A notation that expresses the grammar of a computer language.	*http://en.wikipedia.org/wiki/Backus-Naur_Form*
GEN2PHEN	EU project integrating genotype and phenotype data.	*http://www.gen2phen.org*
GSVML	Genomic Sequence Variation Markup Language	See *Nakaya J, Kimura M, et al. 2010*[16]
HPO	Human Phenotype Ontology	*http://www.human-phenotype-ontology.org/*
Jackson	Java JSON library	*http://wiki.fasterxml.com/JacksonHome*
JAVA	General programming language	*http://www.java.com*
JAXB	Java JSON library	*http://jaxb.java.net/*
JSON	Javascript Object Notation	*http://en.wikipedia.org/wiki/JSON*
LSDB	Gene variant database, Locus Specific Database	*-*
MAGE-TAB	A tab-delimited format for representing functional genomics data.	*http://www.mged.org/mage-tab*
MIRIAM	The MIRIAM Registry provides a set of online services for the generation of unique and perennial identifiers, in the form of URIs.	*http://www.ebi.ac.uk/miriam/main/*
MOLGENIS	Software generating infrastructure (databases, APIs, GUIs) for life science projects.	*http://www.molgenis.org*
Object Model	An abstract representation of a domain’s concepts, data, and relationships between these, used to design or generate software.	-
Observ-OM	A simple system to format and exchange observation data.	*http://www.molgenis.org/wiki/ObservStart*
ORCID	Open Researcher and Contributor Identification	*http://orcid.org/*
PML/DVAR	An implementation of the PaGE-OM object model.	*http://www.openpml.org/*
RelaxNG	Schema definition language for use with XML.	*http://relaxng.org/*
RDF	Resource Description Framework	*http://www.w3.org/RDF/*
Schematron	High-level schema definition language for use with XML.	*http://www.ascc.net/xml/resource/schematron/Schematron2000.html*
SKOS	Simple Knowledge Organization System	*http://www.w3.org/2004/02/skos/*
SO	Sequence Ontology	*http://www.sequenceontology.org/*
UML	Unified Modeling Language	*http://en.wikipedia.org/wiki/Unified_Modeling_Language*
VariO	Variation Ontology	*http://variationontology.org/*
VCF	Variant Call Format	*http://vcftools.sourceforge.net/specs.html*
XGAP	XGAP is an open and flexible object model for xQTL, GWL, GWA and mutagenesis data	*http://www.xgap.org*
XML	eXtensible Markup Language	*http://www.w3.org/XML/*

We began by incorporating previous work on data requirements [6,8,14] and data modelling activities, such as PaGE-OM [15] and its generalization Observ-OM [16], in the design of VarioML LSDBs specification.

VarioML was developed by an international collaboration of variation experts, over a series of workshops organised by the GEN2PHEN project. The design has closely followed the work of Tyrelle and King [17] on the now defunct BSVM standard, where they proposed using semantically well-defined XML and RDF elements for LSDB data integration. VarioML is designed to serve the greater part of LSDB use cases directly, complementing formats such as GSVML [18] and PML/DVAR [19], the latter being an implementation of the PaGE-OM object model [15]. The format is kept consistent with PaGE-OM and Observ-OM by rooting XML element definitions in the same object model. By providing a structured data framework designed close to application domains, VarioML complements tabular data formats such as VCF [20,21] and MAGE-TAB [22], which are designed for high-throughput and manual/spreadsheet-based data handling needs.

The collaboration’s goal was to readdress these requirements by providing simple data structure components for developing use case specific solutions, defined independently using high-level schema definition languages such as Schematron [23]. While it may sound complex, this approach provides the necessary flexibility to serve simple specifications for straightforward use cases, while simultaneously enabling development of more complex specifications, all the while maintaining a common foundation of terms, logic, and tooling that integrates both.

### Ontologies: How much meaning is enough?

Reducing the inherent complexity of annotation formats began by rooting the semantics of the VarioML standard as deeply as possible into base ontologies that underlie science and logic in general. This highlighted the need for a new harmonized model for describing scientific observations in general, providing a common language usable across all domains. For this purpose, a new object model, Observ-OM [16], was developed.

In Observ-OM, four basic concept classes represent all elements of any kind of observable data: Targets, Features, Protocols, and Observations. The value this model represents for the variation pipeline is hard to overstate, as it represents what is probably the maximum possible simplification of elements common to all usable scientific observations regarding variants and associated phenotypes.

**Figure 1:**
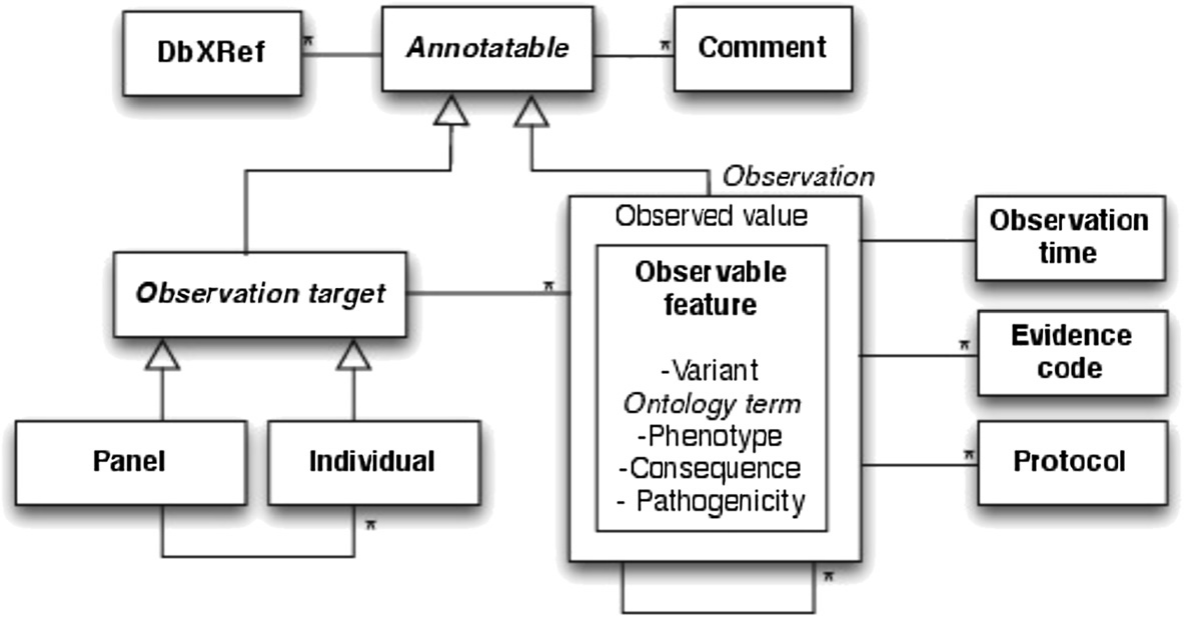
***Simplified conceptual UML object model used in VarioML.*** The VarioML object model is derived from Observ-OM (http://www.observ-om.org/wiki/ObservStart), with some modifications to simplify implementation. E.g., *Observable Feature* (such as *phenotype* or *mutation name*) and *Observed Value* (existence of phenotype or variation) are denormalized into a single XML element. This avoids unnecessary nesting of observation elements which do often have one-to-one relationship, in the XML implementation. Entities are composed into **Observations,** having properties such as *evidence codes*, *observation protocols* and *observation time*. Associations between elements are described as single lines, where an asterisk means a *0-to-many multiplicity* relationship; i.e. *Observation* can have one or many evidence codes. All entities also inherit from Annotatable properties which are needed for database cross references and comments. In this case, the open arrow symbol means inheritance or an *is-a* relationship.

Grounded in Observ-OM, VarioML had the task of adding only what is absolutely necessary to provide an intuitive and ‘decision-free’ path for researchers and clinicians: the shortest possible path from variation data in all its current forms, to a unified representation, distributed globally. At the same time, this shortest path had to be extensible to describe non-minimalistic data as needed (Figure 1).

To achieve this, LOVD-based LSDBs [8,24] were used as a content model, in addition to modelling done in previous work [15,25], and in workshops organized by the GEN2PHEN consortium. This modelling meets the requirements specified previously [26,27]. The specification aims to be minimalistic, but has room for additions where the need arises. Despite this simplification, the underlying base schema can be too verbose for many use cases. Therefore it is important that the schema can be “narrowed”, using separate validation tools for specific cases. This has been done for the Cafe Variome pipeline [28], where separate Schematron [23] rules are used for defining the content. Schematron was chosen because it allows making complex assertions about the content of XML documents, more complex than are possible using the RelaxNG schema language [29] used for defining the base VarioML schema, which has better tooling support for defining initial schema elements.

Existing ontologies, such as the Human Phenotype Ontology (HPO) [30], Sequence Ontology (SO) [31], and Variation Ontology (VariO) [32], can be used with VarioML. Separate SKOS (Simple Knowledge Organization System) [33] vocabularies have been provided for elements which do not have online definitions. The semantics of VarioML elements are well defined via the Observ-OM model; content can be relatively easily transformed to RDF representations for linked data approaches [34]. An example XSLT application is provided for converting Cafe Variome XML content to an RDF schema, derived using the Pharmacogenetics ontology [35].

**Figure 2:**
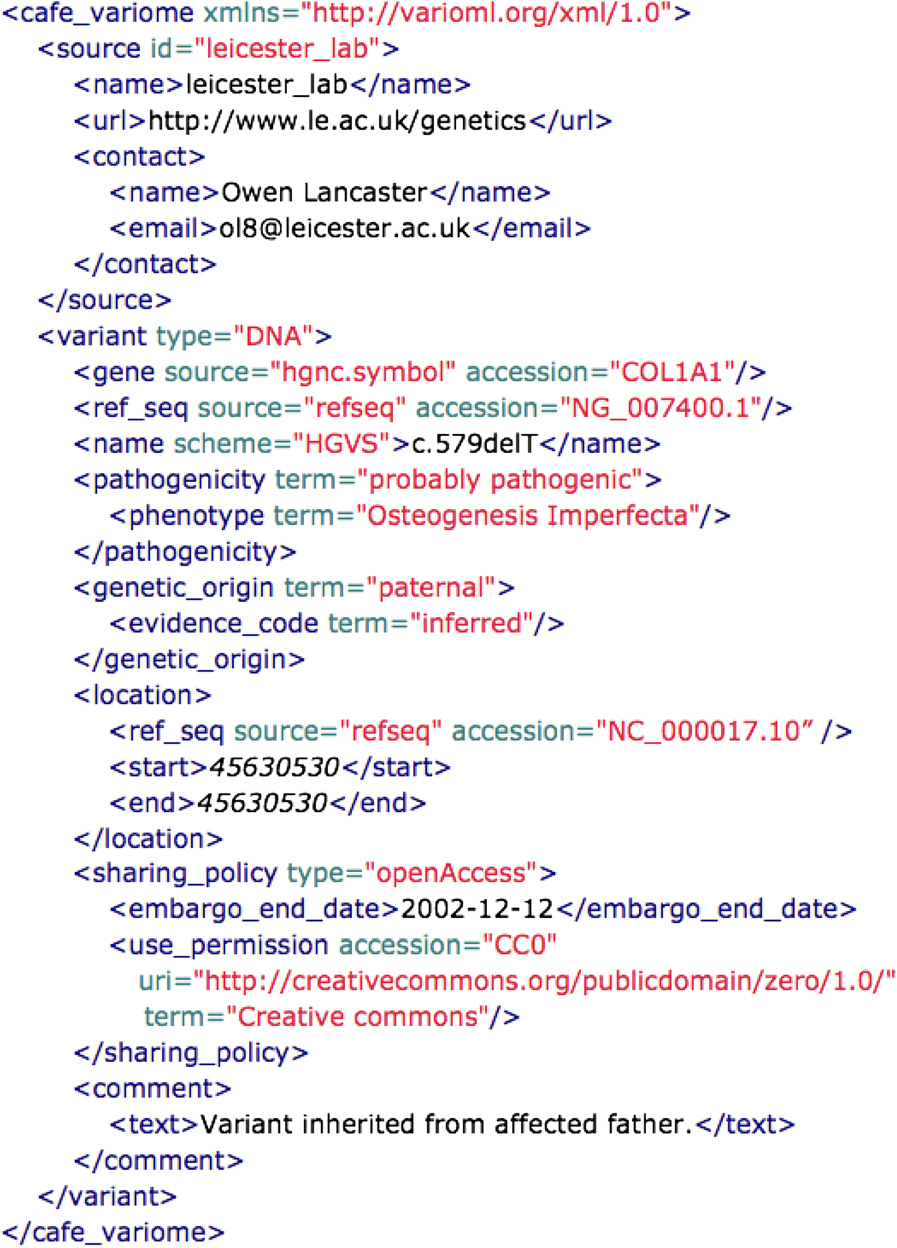
***A Cafe Variome submission of a COL1A1 variant.*** The different VarioML elements of the data submitted are flanked by the corresponding XML tags and explained in the text.

A leading example of a submission tool that fulfills this standard is the Cafe Variome platform [28] for announcing and advertising disease-related variations identified by diagnostic laboratories, allowing them to be shared by diverse third parties. This platform, when integrated with diagnostic software, allows push-button submission of data from tables to central databases. For these submissions, the single variant is the agreed-upon central organizing concept [26]. Variants should be submitted in VarioML format, as seen in the example in Figure 2.

To date, this functionality has been built into the Leiden Open Variation Database [24]; GenSearch [36], a tool to detect and interpret variants in DNA sequences obtained by capillary sequences; BC|SNPMax [37], a data management tool for genomic research; and is currently being testing with Alamut [38].

## Results

### Composing the format

VarioML is composed from an underlying set of XML elements, which reuse the same structural components. Most of the XML elements like *phenotype, consequence and evidence_code* are so-called ontology terms, which have necessary properties for making cross references to existing ontologies in a flexible way:

<phenotype term = "Autoimmune polyglandular syndrome type 1" accession = "240300" source = "omim"/>

All ontology terms can be annotated with comments and database cross-references (see Figure 1). Elements can be extended by adding new schema elements: *Phenotype* is an example of an observation element which reuse properties from the *ontology term* element. Observation elements have additional information related to the observation, such as *date* and *evidence* codes For example, an observed “*consequence of mutation*” has the evidence code “*curator inference,*” as defined in the Evidence Code ontology [39]:

<consequence term = "translational frameshift" accession = "SO:0001210" source = "obo.so">

<evidence_code term = "curator inference" accession="ECO:0000205" source="obo.eco"/>

</consequence>

*Pathogenicity*, on the other hand, is a special case of consequence element, having an optional *scope* attribute for indicating if the variant has been observed in an individual, family, or population. The *pathogenicity* element also has an optional *phenotype* element for specifying causal relationships explicitly, where needed:

<pathogenicity term = "probably pathogenic"

uri = "http://purl.org/varioml/pathogenicity/skos/1.0#p_0003"

scope = "family" >

<phenotype term = "Osteogenesis Imperfecta, Type I"

accession="166200" source="omim"/>

<evidence_code term = "curator inference" accession="ECO:0000205" source="obo.eco"/>

</pathogenicity>

VarioML is currently used as the XML data submission and release format for the Cafe Variome announcement service. An example of this implementation is given in Figure 2.

In the next section, we provide a brief overview of the elements seen in Figure 2, an example of the straightforward variant descriptions that make up the bulk of LSDB submissions.

### Modular elements for variant annotation

To match raw variation data to the standard descriptions specified in *‘Guidelines for establishing locus specific databases’*[26], users simply match their data to VarioML elements. For large data sets, VarioML’s validation tools can be used to check converted data. Following is a partial list of variant data elements required and validated by VarioML, some of which are used in Figure 2.

### Source

The *source* element stores information on the submitting sources, with attributes for submitting *instance* or *database*, *contact* details, and *acknowledgements*.

VarioML requires submitter identification using the db_xref element, and recommends that an ORCID ID [40] be obtained for this purpose. ORCID (Open Researcher and Contributor Identification) is a platform building towards automation of authorization and access infrastructure for institutions and federations [41]. This combination of standardization of data and researcher ID are necessary components of a translational information system, in which data discovery, access, and incentives to sharing must be closely integrated, constituting a sustainable ecosystem [42].

### Variant

The *variant* element can be used in a straightforward manner, bounding information reported on a variant described using the HGVS naming scheme [43], which has recently been formally described as a scientific sub-language in Extended Backus-Naur Form [44].

Important*Variant* also provides recursive sub-elements, for cases where the reporting variant is composed of other variants located on the same or a sister chromosome.

*Variant* has an optional *observation target* attribute. For simplicity, the *Panel* element is used as a generic target for *variant*: *panel* can be used to describe any number of individuals, with or without group-specific identifiers, such as *family* or *population*.

### Gene

The *Gene* is given as a database cross-reference, where *source* indicates the database or system (e.g. HUGO), and 'accession' is the gene name (e.g., AGA). HGNC symbols or IDs [45] must be used for the primary name of a gene. Gene is a *database cross-reference* type, which is conceptually similar to *ontology term*.

When specifying sources, the MIRIAM namespace identifiers should be used [46]. For example, the identifier for HGNC gene symbols is *hgnc.symbol*:

<gene source = "hgnc.symbol" accession = "COL1A1"/>

**Figure 3:**
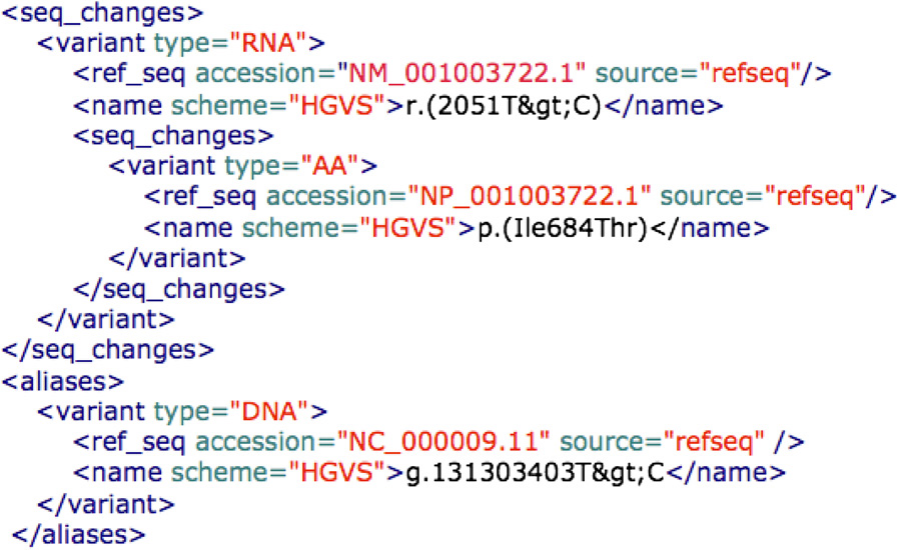
***VarioML elements extending the core schema*. **The VarioML elements describing the effect of an AIRE variant at the transcript and protein levels are flanked by the corresponding XML tags and explained in the text.

Use of database identifiers specified in the MIRIAM registry insures consistent naming of sources [47]. Examples of MIRIAM in use are given in Figures 2,3.

### Reference sequence

Variants must always be submitted in the context of a *reference sequence*. LRGs are the preferred form for reference sequences [48]. LRG sequences ‘provide a stable genomic DNA framework for reporting variations with a permanent ID and core content that never changes’ [49].

### HGVS name

The *name* element gives the variant name. While *name* has an optional attribute *scheme* for indicating the naming scheme used, the primary name of a variant must be given using the HGVS naming scheme [43,44]. To allow machine-processing, the “>” character in an HGVS name must be encoded to “&gt;”, as defined in the XML specification [50].

### Pathogenicity

*Pathogenicity* has values such as: *No known pathogenicity, Probably not pathogenic, Unknown, Probably pathogenic,* and *Pathogenic.* These values meet the guidelines for reporting unclassified variants established in 2007 [51]. These and alternative terms are provided in a separate SKOS vocabulary [52].

### Genetic origin

The *genetic origin* of a variation can be given in its own observation element. The vocabulary defined in the VarioML SKOS vocabulary can be used [53].

### Location

A variant can have multiple locations defined on different reference sequences. The *location* element provides precise standardized positioning of variants, giving possibility to integrate data easily with DAS services [54] and genome browsers. In Figure 2, the variant position is given using chromosomal coordinates.

### Sharing policy

The inclusion of the *sharing policy* element in VarioML allows setting fine-grained access control policies per individual variant. Possible values are *closedAccess, embargoedAccess, restrictedAccess* and *openAccess,* which are defined in the OpenAIRE guidelines [55]. *Embargo end date* tells when data can be publicly released. *Use permission* is an ontology term which can be used for citing licensing terms. The vocabulary describing these policies is taken from the OpenAIRE specification [56].

### Additional XML elements

Additional elements, shown in Figure 3, demonstrate a first tier of extensions of the core specification. The following elements are not yet implemented in applications, and may be redefined and modified later according to community needs.

### Effects on RNA and AA sequences

Effects on gene products can be given under the *seq_changes* observation element, which can store information on RNA and AA sequences in a recursive manner, using nested *seq_changes* elements. For example, a top-level *variant* element specifies a unique position on the genome, which can contain RNA level variants in a *seq_changes* sub-element, which in turn can contain corresponding AA changes in a further nested *seq_changes* sub-element. *Consequence* annotations can be assigned on these different levels, representing expert agreement about which level is causative of a given consequence.

The *Variant* element also has places for *aliases* and *haplotype sets*. *Aliases* are for legacy annotations and variations which have been named using different reference sequences. *Haplotypes* are sets of variants which are in *cis* relative to one another. These elements can be used if the main variant represents a larger sequence region containing multiple variations. Implementation of these extensions will be finalised as more experience is gained in handling such variations.

### Frequency

Variants can have one or more frequency elements, each of which can use one of three formats: decimal number, number of cases, or categorized value. The decimal number type gives frequency as a floating point value; number of cases type gives frequency as a count; and the categorized value gives frequency as an ontology term, for categorized observations such as “exists” or “less than 100”. *Population, evidence ontology term, evidence code, protocol id* and *comment* attributes provide context for the frequency value.

### Implementation

XML remains the reference platform of choice, providing a mature specification, and advanced tools such as schema definition languages [57]. Our use of extensible XML elements encourages implementers to collaborate closely, since extending the format requires formulating a common development strategy. However, we realized that, as the XML schema is extended, a lacuna could easily open between the data model and its implementations. Adapting changing XML schema into applications has tended to be laborious. We reasoned that absolving application developers of the need to reinvent the wheel of data translation across formats was fundamental to easing the effort and cost of adopting the VarioML standard. We further reasoned that, in biomedicine as well as in other scientific domains, the era of big data likely makes it no longer feasible to develop formats separately from the tooling that transports them bidirectionally across the required data languages (recalling the computer science maxim, ‘Data = code’ [58][59]).

In practice, this meant that VarioML schema elements have to work transparently as XML, JSON, and possibly in future as RDF, *without incurring a cost of translation to the implementer or user*. Providing support for bidirectional translation to JSON was a clear way in which we could enable schema extensions to much more quickly and inexpensively be reflected in applications. To this end, VarioML comes with Java and JSON APIs (application programming interfaces), which developers can plug into their applications to handle conversion and publication.

**Figure 4:**
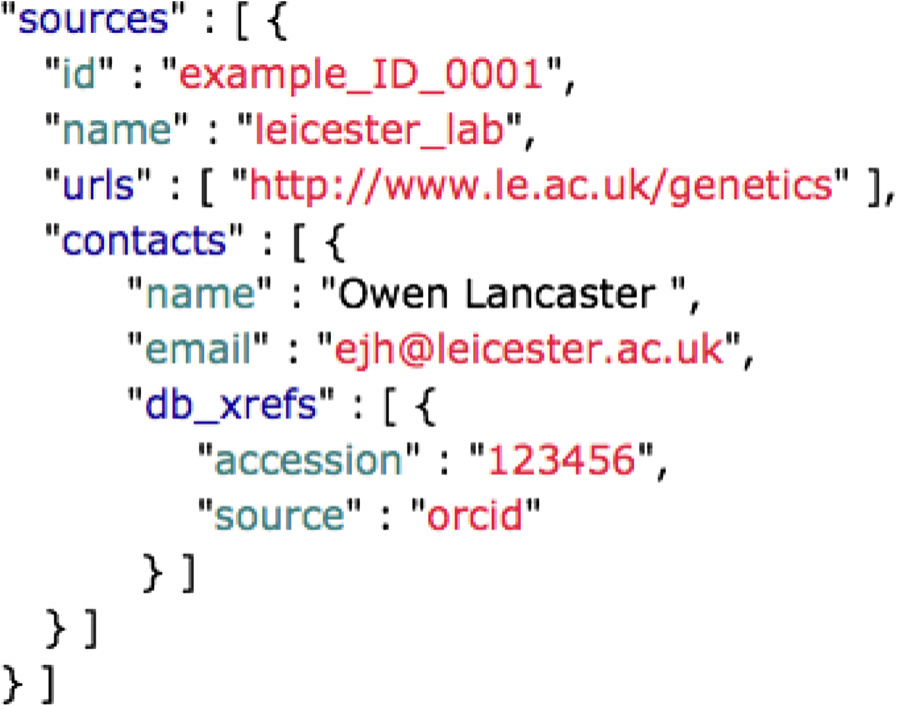
***VarioML in JSON format.*** XML elements are mapped to JSON objects using JAXB and Jackson annotations via VarioML's Java API. Repeating XML elements become pluralised into JSON arrays. Because JSON does not have an equivalent to XML attributes, XML attribute names can clash with inner element names. In these cases, the JSON name for the XML attribute is changed. Otherwise, mapping VarioML from XML to JSON is a direct transformation of the data structure.

JSON is the common data serialization format now recognized as the *lingua franca* for data exchange over the web (while we find no academic reference to this fact, it is a commonplace in the web application domain. e.g. [60]), proven to be faster and consume fewer resources than XML [61]. Serializing to JSON facilitates applying programming techniques to data, to create interactive content, user interface components, etc. in formats native to the web, simplifying the provision of data access [62]. VarioML provides a JSON implementation, currently defined using JAXB [63] and Jackson [64] annotations. This JSON implementation is made available as a VarioML Java library [65], which can be used to read and write XML and JSON versions of the format. An API is auto-generated from XML instances, providing Java object representations for all VarioML objects. This API will be kept synchronized with the format, and can be used as a helper tool in Java applications. A JSON example of the source element is shown in Figure 4.

In addition to JSON support, the VarioML Java API supports EXI, a binary compression of XML. EXI support leverages the VarioML XML schema, reducing file sizes and the time required for data processing operations by factors of three to ten [66]. While VarioML is primarily focused on curated variant entries produced at the end of HTP pipelines, the use of EXI makes it feasible to use VarioML for earlier stages of production pipelines.

The JSON tooling provided with VarioML makes it possible for implementers to develop dynamic user interfaces with substantially less effort and cost [67], expanding on the possibilities demonstrated by the *‘Web Analysis of the Variome’* project [68,69]. A logical next step to contribute to the end-to-end variome pipeline would be to build a variant annotator widget, usable with different database implementations.

## Discussion

VarioML has been designed to immediately serve data exchange needs for LSDB’s, focusing on curated variant entries produced at the end of data production pipelines. However, as an end-to-end variation pipeline comes together, for a common specification to be truly useful, it must be extensible beyond the immediate LSDB use-case. Next-generation sequencing pipelines make possible exon-capture scenarios in which tens to hundreds of patients are sequenced in one or more genes, presenting new challenges in variant calling, annotation, and data sharing [70]. To meet the holistic data integration challenge and realize the grand variation pipeline, we need to harmonize the data models, data standards, and content specifications in use at each step, to encompass the descriptive needs of all LSDBs, standardizing their quality and accuracy, and enabling more comprehensive and high quality data curation [26]. A number of projects have previously attempted to fill these requirements and provide a single multipurpose implementation format for LSDBs, yet have come up against difficulties at multiple levels of design and implementation [11]. Variation data can be arbitrarily complex, making a single standard specification elusive. LSDB use cases vary a great deal in the depth of detail and structure needed for data capture. Complex standards have proven too time-consuming to implement. Solutions designed in one format cannot be readily transferred to another. Further hampering progress towards a common specification are the multiple strong motivations which labs have to keep variant data private [71].

As work on a unified variome progresses, genetics research faces a paradox: another attempt at a variation standard will not be enough to surmount these obstacles. No matter how comprehensive our current efforts, new standards will inevitably follow. Our understanding of genomic variation is rapidly evolving; multiple and often conflicting forms of variant annotation seem to be required to serve differing use cases, implementations, and viewpoints. Attempts to comprehensively integrate all such descriptions in a single standard can, at this point, be expected to produce either unmanageable complexity, or inaccurate oversimplification. Furthermore, looking ahead, we can be fairly certain that new discoveries and technologies will arise that cannot be presently designed for.

In designing VarioML, we therefore turned away from seeking a top-down monolithic solution, choosing instead to make a lightweight framework for composing interoperable, use-case specific ‘micro-standards’ around the generalized concepts of *observation targets, ontology terms,* and *observations,* adapted from the Observ-OM specification [16]. The core set of VarioML schema elements can be used as building blocks, addressing use cases from the most minimal towards the more complex, while maintaining the underlying interoperability of the data. Implementations can use as many or as few of these blocks as needed, and new elements can be added into the specification as needed. However, with this extensibility also comes the danger of the fragmenting specifications into incompatible versions. While elements are utilized in an increasing number of new representations and schema, at the same time, they are also converging all variation data into a unified variome pipeline. Yet equalizing these needs for divergence and convergence is a task that cannot be planned by a committee. As next generation sequencing continues accelerating both the scale and complexity of the data produced, all producers of variation data have a stake in decreasing the gap between a general variation annotation standard, and the community it serves. Accordingly, VarioML is intended less as a ‘completed’ specification, more as a nucleation centre around which new specifications can be developed. All variation data producers are called upon to develop this specification collaboratively.

To this end, VarioML development has been turned over to the community. The specification lives inside an open collaboration framework, tightly binding new variation reporting structures to the common schema and tooling, maintaining consistent application generation capability and backwards compatibility with earlier applications and data [72]. We chose two forums to realize this collaboration framework: the VarioML forum at the science-centered GEN2PHEN Knowledge Center [73], where format details are discussed alongside immediate access to a unified catalog of LSDBs and other tools for variation data integration; and VarioML’s GitHub repository [74], where the schema and XML, JSON, Java, and RDF tools are available, in addition to UML documentation that clarifies the relationships between specification and implementations [75]. Modified or new compositions using schema elements must be reported in either of these forums and discussed openly, enabling the collaborative extension of the format without breaking existing implementations.

The open-ended nature of the VarioML specification means there should continuously be elements under active redefinition and modification by the community. These features should not be implemented in applications until consensus on usage is reached. For example, the *Variant* element allows recursive sub-elements, for cases where the reporting variant is composed of other variants located on the same or a sister chromosome. Yet this and other features (see *Additional XML elements* section) are not currently implemented in applications.

## Conclusions

VarioML enables researchers, diagnostic laboratories, and clinics to improve the quality of human variation information, and to share that information with ease, clarity, and without ambiguity. VarioML resolves the inherent tendency of variation data to diverge in format and meaning through a modular design that lives in an open collaboration framework, composed of two linked community forums.

With this open collaboration framework, the variome community itself closely binds the evolution of the annotation format and its tooling to the science of the study of human mutations. For example, as new configurations and extensions of the format are developed by various implementers, they can be discussed and improved at the GEN2PHEN Knowledge Center, alongside submissions of relevant data made through Cafe Variome. As community consensus emerges, this agreement translates to changes in the schema and tooling in the common repository. At each step, the provenance of even small contributions are captured and can be used as microattributions [42].

For such bottom-up, self-organizing management of a common variation standard to work, teams working at critical junctions in the variation pipeline must translate a passion for the vision of the unified variome, into both implementation and development of the shared standard. To date, VarioML has been implemented in three applications (the Leiden Open Variation Database [24], GenSearch [36], and BC|SNPMax [37]), and is currently being tested in a fourth (Alamut [38]). In each case, VarioML is used to enable push-button submission of data through the Cafe Variome service [28].

With consensus on a minimal standard, implementation is the remaining bottleneck. Users, from research teams to commercial software producers, need to focus their software-related activity to those tasks in which their resource costs are proportionally smaller than the added value afforded by adopting new tools and data models. VarioML has been designed to minimize the effort required for both implementation and extension, framing the specification itself with Java and JSON APIs on the one hand, and an open collaboration framework on the other. We hope this approach proves useful throughout the variation science community, as it meets the challenge and potential of next generation sequencing, and quickens to open the path from discovery to delivery.

## Competing interests

The authors declare no competing interests.

## Authors’ contributions

MB collated the contributions of other authors, and wrote the body of the manuscript. IFACF was a central participant in defining and refining the VarioML format, as a participant in GEN2PHEN and as one of the creators of the Leiden Open Variaton Database. OL was a central participant in defining and refining the VarioML format, as a participant in GEN2PHEN and as one of the creators of the Cafe Variome data submission platform. TA participated in defining and refining the VarioML format. AAB participated in refining the VarioML format, as an implementer of the specification in the BC|SNPMax application. DA participated in refining the VarioML format, as an implementer of the specification in the Gensearch application. CB participated in defining and refining the VarioML format. MC participated in defining and refining the VarioML format. RD participated in defining and refining the VarioML format. AD participated in defining and refining the VarioML format. GP participated in defining and refining the VarioML format, as a participant in GEN2PHEN and representing the National Ethnic Mutation Databases in this activity. MS was a central participant in defining and refining the VarioML format, as a participant in GEN2PHEN and as one of the creators of Observ-OM, Pheno-OM, and the Molgenis application platform. PEMT was a central participant in defining and refining the VarioML format, as a participant in GEN2PHEN and as one of the creators of the Leiden Open Variaton Database. GT was a central participant in defining and refining the VarioML format, as a participant in GEN2PHEN and as one of the creators of the ORCID researcher identification platform. MV was a central participant in defining and refining the VarioML format, as a participant in GEN2PHEN and as the creators of the Varioation Ontology. AB was a central participant in defining and refining the VarioML format, as chair of GEN2PHEN and as one of the creators of the Cafe Variome data submission platform. JM was a central participant in defining and refining the VarioML format, as a participant in GEN2PHEN and as the managing creator of the VarioML specification. All authors participated in the design and testing of VarioML. All authors read and approved the final manuscript.
